# Furanodiene Induces Extrinsic and Intrinsic Apoptosis in Doxorubicin-Resistant MCF-7 Breast Cancer Cells via NF-κB-Independent Mechanism

**DOI:** 10.3389/fphar.2017.00648

**Published:** 2017-09-14

**Authors:** Zhang-Feng Zhong, Hai-Bing Yu, Chun-Ming Wang, Wen-An Qiang, Sheng-Peng Wang, Jin-Ming Zhang, Hua Yu, Liao Cui, Tie Wu, De-Qiang Li, Yi-Tao Wang

**Affiliations:** ^1^Guangdong Key Laboratory for Research and Development of Natural Drugs, School of Public Health, Guangdong Medical University Zhanjiang, China; ^2^State Key Laboratory of Quality Research in Chinese Medicine, Institute of Chinese Medical Sciences, University of Macau Macao, China; ^3^Division of Reproductive Science in Medicine, Department of Obstetrics and Gynecology, Feinberg School of Medicine, Northwestern University, Chicago IL, United States; ^4^Center for Developmental Therapeutics, Chemistry of Life Processes Institute, Northwestern University, Evanston IL, United States; ^5^Department of Pharmacy, The Second Hospital of Hebei Medical University Shijiazhuang, China

**Keywords:** furanodiene, multidrug resistance, breast cancer, cell apoptosis, intrinsic/extrinsic apoptosis, NF-κB

## Abstract

Chemotherapy is used as a primary approach in cancer treatment after routine surgery. However, chemo-resistance tends to occur when chemotherapy is used clinically, resulting in poor prognosis and recurrence. Currently, Chinese medicine may provide insight into the design of new therapies to overcome chemo-resistance. Furanodiene, as a heat-sensitive sesquiterpene, is isolated from the essential oil of *Rhizoma Curcumae*. Even though mounting evidence claiming that furanodiene possesses anti-cancer activities in various types of cancers, the underlying mechanisms against chemo-resistant cancer are not fully clear. Our study found that furanodiene could display anti-cancer effects by inhibiting cell viability, inducing cell cytotoxicity, and suppressing cell proliferation in doxorubicin-resistant MCF-7 breast cancer cells. Furthermore, furanodiene preferentially causes apoptosis by interfering with intrinsic/extrinsic-dependent and NF-κB-independent pathways in doxorubicin-resistant MCF-7 cells. These observations also prompt that furanodiene may be developed as a promising natural product for multidrug-resistant cancer therapy in the future.

## Introduction

Multidrug resistance (MDR) is one of the major factors of chemotherapy failure in the clinic, since it predicts poor prognosis and cancer recurrence of patients. Cancer drug resistance includes primary resistance before chemotherapeutic treatment and acquired resistance after chemotherapeutic exposure (Chemo-resistance). A MDR phenotype is defined as the resistance of malignant cells to different drugs with diverse structures and functions after exposure to the drug (Biedler and Riehm, 1970; Kaye, 1988; Vtorushin et al., 2014; Wu et al., 2014). Since a significant proportion of multidrug-resistant cancer cells could survive even after chemotherapy that results in chemo-resistance, disease progression, and recurrence.

Furanodiene is a heat-sensitive sesquiterpene found in the essential oil of *Rhizoma Curcumae* (Baldovini et al., 2001). In our previous study, furanodiene was confirmed to inhibit breast cancer growth (Zhong et al., 2012a) through altering angiogenesis (Zhong et al., 2012b) and energy metabolism (Zhong et al., 2016b), suppressing migration and invasion (Zhong et al., 2014), and enhancing the anti-cancer effect of doxorubicin and tamoxifen (Zhong et al., 2012c, 2016a) *in vitro* or *in vivo*. Additional researchers have also elucidated that cell death was induced by furanodiene in HepG2 cells (Xiao et al., 2007), HL60 leukemia cells (Ma et al., 2008), 95-D lung cancer cells (Xu et al., 2014), and uterine cervical cancer cells (Sun et al., 2009).

Apoptosis is known as a physiological process of cell deletion and is also a process of programmed cell death. It is stimulated by external or internal events of cells (Strasser et al., 2000), namely, the extrinsic pathway mediated by the death receptor and the intrinsic pathway mediated by mitochondria. The death receptors include tumor necrosis factor (TNF) receptors and TNF-related apoptosis-inducing ligand (TRAIL) receptors. As a surface receptor, TNF receptor-1 (TNF-R1) interacts with TNF and forms a receptor-proximal complex, which recruits a series of downstream factors, including Caspase-8, IκB kinase (IKK) α and β, resulting eventually in NF-κB activation (Chen and Goeddel, 2002; Reed, 2003). Otherwise, the mitochondrial pathway involves the Bcl-2 protein family and Caspase activation (Strasser et al., 2000; Czabotar et al., 2014). Among these effectors, Caspase-8 is an initiator caspase and downstream effector of TNF receptors and TRAIL receptors, directly cleaving Caspase-3/7 to propagate apoptosis signaling. Poly (ADP-ribose) polymerase (PARP) is the main cellular substrate of Caspase-3/7 for cleavage (Fulda and Debatin, 2006).

Even though growing evidence claims that furanodiene possesses anti-cancer activities in various types of cancers, the underlying mechanisms against chemo-resistant cancer are not fully clear. The present study aims to evaluate the initial effects of furanodiene on cell apoptosis and the underlying mechanisms in doxorubicin-resistant MCF-7 human breast cancer cells.

## Materials and Methods

### Chemicals and Reagents

Furanodiene (FUR) was purchased from the National Institutes for Food and Drug Control. The Roswell Park Memorial Institute-1640 (RPMI-1640) was used as the culture medium and was purchased from Gibco (Gaithersburg, MD, United States). Fetal bovine serum (FBS), phosphate-buffered saline (PBS), penicillin-streptomycin (PS), and 0.25% (w/v) trypsin/1 mM EDTA were obtained from Invitrogen (Carlsbad, CA, United States). 3-[4, 5-Dimethyl-2-thiazolyl]-2, 5-diphenyltetrazolium bromide (MTT), 2′, 7′-dichlorodihydrofluorescein diacetate (H_2_DCF-DA), CellROX^®^Deep probe, propidium iodide (PI), and Annexin V/PI detection kit were obtained from Molecular Probes (Eugene, OR, United States). Doxorubicin (DOX) and *tert*-Butyl hydroperoxide (TBHP) solution were supplied by Sigma-Aldrich (St. Louis, MO, United States). TNF-α immunoassay kit was purchased from R&D Systems (Minneapolis, MN, United States). Radioimmunoprecipitation assay (RIPA) lysis buffer and primary antibodies against TNF-R1 and p65 were obtained from Santa Cruz (Santa Cruz, CA, United States). Primary antibodies against p-IKKα/β (Ser176/180), IKKα, IKKβ, Bcl-xL, Bax, Bad, Caspase-7, Caspase-8, PARP, GAPDH, and β-actin, as well as the secondary antibodies were purchased from Cell Signaling (Danvers, MA, United States). siRNA was purchased from Santa Cruz Biotechnology (Santa Cruz, CA, United States).

### Cell Culture and Drug Treatment

Doxorubicin-resistant MCF-7 breast cancer cells were cultured as previously reported (Zhong et al., 2016b). The stock solutions of furanodiene (100 mM) and Doxorubicin (2 and 100 mM) dissolved in DMSO were diluted to different concentrations as needed.

### Cell Viability Assay

Cell viability was performed by MTT assay as described previously (Zhong et al., 2011). Briefly, exponentially growing cells were seeded in 96-well plates at a corresponding density depending on different cell lines. Following the required incubation period, cell viability was determined by adding 100 μL of MTT (1 mg/mL). The absorbance values at 570 nm were recorded using SpectraMax M5 microplate reader (Molecular Devices, Silicon Valley, CA, United States).

### Cell Cytotoxicity Assay

LDH release from the cells was measured with a cytotoxicity detection kit (Roche), according to the manufacturers’ protocol. In brief, supernatants (70 μL) were dispensed to a new 96-well plate. A 50-μL volume of supernatants was taken from each well for the reaction with LDH substrate after centrifugation at 350 *g* for 5 min. The absorbance values at 490 and 600 nm were recorded using SpectraMax M5 microplate reader (Molecular Devices, Silicon Valley, CA, United States).

### Colony Formation Assay

Cells were seeded in 6-well plates at a density of 5 × 10^2^/well. After a 15-day incubation at 37°C for visible colonies, these colonies were fixed with 4% (w/v) paraformaldehyde for 15 min and stained with crystal violet for 5 min. The images with colonies (≥50 cells as a colony) were captured using a microscope (Olympus MVX10, Japan) equipped with a digital camera (ColorView II, Soft Imaging System, Olympus).

### CFDA-SE Cell Proliferation Assay

Cell proliferation determination was conducted with the CFDA-SE probe. Briefly, cells were seeded in 6-well plates at a density of 5 × 10^2^/well and stained with CFDA-SE probe according to the manufacturer’s protocol. Then the cells were harvested and washed with PBS following drug treatments as required for 6 days. CFDA-SE fluorescence was detected using flow cytometry (BD FACS Canto^TM^, BD Biosciences, San Jose, CA, United States) and represented using FlowJo software (TreeStar, Ashland, OR, United States).

### JC-1 Assay

Mitochondrial membrane potential (ΔΨ*m*) was performed by JC-1 assay according to the previous report (Zhong et al., 2012a). Briefly, cells were cultured in 6-well plates at a density of 2 × 10^5^/well and in black 96-well plates (with transparent bottom) at a density of 2 × 10^4^/well, respectively. The incubation periods were performed as required. Then the culture medium was removed and the cells were washed twice with PBS. A further incubation with loading dye buffer (containing 2.5 μg/mL JC-1 and 10 mM glucose) was performed for 15 min at 37°C. The cells were harvested and mean fluorescence intensity of FITC was detected using a flow cytometry (BD FACS Canto^TM^, BD Biosciences, San Jose, CA, United States). Furthermore, JC-1 fluorescence was also observed via fluorescent microscopy and the images were captured using an Axiovert 200 fluorescent microscope (Carl Zeiss) and AxioCam HRC CCD camera (Carl Zeiss).

### Caspase Activity Assay

Caspase-Glo assay kit (Promega, Madison, WI, United States) was used to determine caspase activity according to the manufacturer’s instruction. After indicated treatments, culture medium (50 μL) was discarded. Subsequently, caspase assay reagent (50 μL) was added to each well. Incubation protected from light under shaking condition lasted for 30 min. Reaction complex (70 μL) was transferred to white-walled 96-well plates. The luminescence was measured using SpectraMax M5 microplate reader (Molecular Devices, Silicon Valley, CA, United States). Caspase activity was calculated as the percentage of control. All samples were repeated in triplicate.

### Western Blotting Assay

Western blotting assay was performed according to the previous studies (Zhong et al., 2012b; Quan et al., 2015). Briefly, cells were harvested and the total proteins were extracted with RIPA lysis buffer after the required treatments. Equal amounts of total proteins were separated by appropriate SDS-PAGE followed by transferring onto a PVDF membrane. After blocking with non-fat milk, the membrane was incubated with specific primary antibodies and the corresponding second antibodies, respectively. The specific protein bands were visualized with an Amersham^TM^ ECL^TM^ advanced western blotting detection kit (GE Healthcare Life Sciences, United Kingdom).

### Annexin V/PI Staining Assay

Cells were seeded in 6-well plate at a density of 2 × 10^5^/well and allowed to adhere overnight. Treatment and incubation period were performed as required. Cell apoptosis was determined by Annexin V/PI labeling according to the manufacturer’s protocol (Invitrogen). The early and late apoptotic cells were detected using a flow cytometry (BD FACS Canto^TM^, BD Biosciences, San Jose, CA, United States) based on the Annexin V and PI staining.

### Cellular Reactive Oxygen Species (ROS) and Calcium Generation Assay

Cells were seeded in 6-well plates at a density of 2 × 10^5^/well or in black 96-well plates (with transparent bottom) at a density of 2 × 10^4^/well, respectively. Then the cells were incubated in a humidified incubator for 24 h. The culture medium was replaced with FBS-free RPMI-1640 medium containing 2 μM H_2_DCF-DA, CellROX^®^ Deep Red probe, or Fluo-4 AM with a 30-min incubation at 37°C to assess the reactive oxygen species (ROS) or calcium levels. Cellular ROS and calcium generation was determined after the drug treatment as required. TBHP treatment was used as a positive control. For quantitative assessment, the cells were collected, re-suspended in PBS and analyzed using flow cytometry (BD FACS Canto^TM^, BD Biosciences, San Jose, CA, United States). The mean fluorescence intensity of 10,000 analyzed cells (corrected for autofluorescence) of each group was measured for the total ROS or calcium generation, and represented by FlowJo software (TreeStar, Ashland, OR, United States). For direct observation assessment, images were captured using high-content imaging.

### Quantitative Immunoassay for TNF-α Release

Cells were seeded in 12-well plates at a density of 1 × 10^5^/well. The conditioned culture medium was collected and the TNF-α release from the cells was determined by immunoassay kit (R&D Systems, Minneapolis, MN, United States) according to the manufacturer’s protocol. The absorbance values at 450 and 570 nm were determined using SpectraMax M5 microplate reader (Molecular Devices, Silicon Valley, CA, United States).

### TNF-α-TNF-R1 Binding ELISA

The binding assay was performed according to the previous report (Leung et al., 2012). Briefly, microtiter plates were coated with TNF-α (0.625 μg/mL) in PBS at 4°C overnight. After washing three times with PBS/0.05% Tween 20 (PBST), the wells were blocked with PBST containing 1% BSA (200 μL) for 1 h. A series of concentrations of the test drugs were added into the wells followed by a 20-min incubation. TNF-R1 solution in PBS (50 μL, 0.2 μg/mL) was added into the wells with further incubation for 2 h. The washed wells were incubated with TNF-R1 antibody solutions (1:1000) in 100 μL of PBST containing 1% BSA for 2 h. Then, the washed wells were incubated with horseradish peroxide-conjugated secondary antibody for 2 h. Finally, the washed wells were incubated with TMB solution (100 μL), followed by quenching treatment with stop solution (100 μL, 2*N* sulfuric acid). The absorbance was measured at 450 nm with SpectraMax M5 microplate reader (Molecular Devices, Silicon Valley, CA, United States).

### Cell Density Assay

After the administered drug treatments, cell density was observed and the images were captured using a microscope (Olympus MVX10, Japan) equipped with a digital camera (ColorView II, Soft Imaging System, Olympus), to survey cell density under 100× magnifications. The representative images were from at least three independent experiments.

### Dual-Luciferase Reporter Assay

Cells were seeded in a 24-well plate at a density of 5 × 10^4^/well. The cells were co-transfected with 0.8 μg pNF-κB-luc and 0.8 μg pRL-TK as a transfection efficiency control. The plasmids and Lipofectamine agent were diluted in Opti-MEM serum-free medium according to Lipofectamine DNA transfection reagent protocol. The diluted DNA was mixed together with diluted Lipofectamine agent at the ratio of 1:1 followed by a 20-min incubation at 25°C. DNA-Lipofectamine (100 μL) complexes was transferred to each well. After a 4-h incubation, the cells were cultivated with fresh completed medium for 48 h. Cell lysates were collected by using passive lysis buffer according to the dual luciferase assay protocol (Zhong et al., 2015). Sample light output was recorded by using SpectraMax M5 microplate reader (Molecular Devices, Silicon Valley, CA, United States). Data were aligned to pRL-TK values prior to normalization with its control.

### Transient Transfection of siRNAs

RNA interference assay was performed using Lipofectamine 2000 agent according to the manufacturer’s protocol. Briefly, cells were seeded in 6-well plates at a density of 2 × 10^5^/well overnight. siRNA and Lipofectamine agent were diluted in Opti-MEM reduced serum medium and mixed gently, respectively. Then, the siRNA-lipofectamine mixtures were transferred to the culture wells, following a 20-min incubation at room temperature. After a 4-h transfection, the cells were refreshed with completed medium. The transfected cells were selected for the further experiments after a 48-h stable incubation.

### Statistical Analysis

All data represent the mean of three separately performed experiments, plus or minus standard deviation or standard error of the mean. The significance of intergroup differences was evaluated by one-way ANOVA using the GraphPad Prism software (GraphPad Software, United States). Newman–Keuls multiple comparison tests were performed for *post hoc* pairwise comparisons. *P*-values less than 0.05 were considered as significant.

## Results

### Furanodiene Displayed Anti-cancer Effects through Inhibiting Cell Viability, Inducing Cell Cytotoxicity, and Suppressing Cell Proliferation in Doxorubicin (DOX)-Resistant MCF-7 (MCF-7/DOX^R^) Cells

**FIGURE 1:**
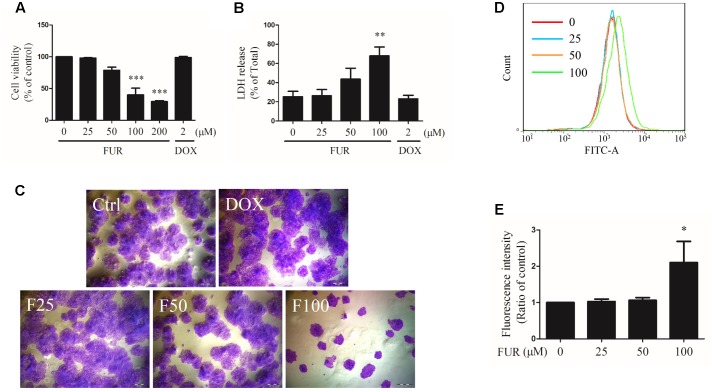
Effect of furanodiene (FUR or F) on cell viability, cytotoxicity, and proliferation in doxorubicin (DOX)-resistant MCF-7 breast cancer cells. Cells were treated with furanodiene (0–200 μM) or doxorubicin (2 μM) for 24 h. **(A)** Cell viability was tested by MTT assay. The values were represented as percentage of the vehicle control. **(B)** LDH release was detected using a commercial kit. The values were represented as percentage of the total LDH release. **(C)** Cells were treated with furanodiene (0–100 μM) or doxorubicin (2 μM) for 24 h. Then the cells were harvested, and cultured in complete growth medium at a density of 200 cells per well. After a 2-week incubation, the fixed cells were stained with crystal violet. The colony formation status was visualized at 200× magnifications with an AxioCam HRC CCD phase contrast microscope. **(D)** Cells were stained with CFDA-SE and then treated with furanodiene (0–100 μM) for 6 days. CFDA-SE fluorescence was detected using flow cytometry. FlowJo software was used for the analysis of flow cytometry data. **(E)** Statistical result of **(D)**. Ctrl stands for control. Data are expressed as mean ± SEM. ^∗^P < 0.05, ^∗∗^P < 0.01, and ^∗∗∗^P < 0.001 vs. control.

As shown in **Figure 1A**, furanodiene inhibited the viability of MCF-7/DOX^R^ human breast cancer cells in a dose-dependent manner. In addition, compared with the vehicle control group, LDH releases from MCF-7/DOX^R^ cells were increased dose-dependently after 24-h treatment with furanodiene (**Figure 1B**). However, neither the cell viability nor the cell cytotoxicity was significantly changed after doxorubicin (2 μM) treatment.

Cell proliferation induced by furanodiene was assessed by colony formation assay and CFDA-SE assay. In colony formation assay, colony formation was strongly inhibited by furanodiene (100 μM) treatment, and there were almost no alterations observed in doxorubicin (2 μM) treatment compared with vehicle control in MCF-7/DOX^R^ cells (**Figure 1C**). After furanodiene (25, 50, and 100 μM) treatment for 6 days, the mean fluorescence intensity induced by furanodiene (100 μM) was stronger than the vehicle control, indicating that furanodiene significantly suppressed the proliferation at high concentration in MCF-7/DOX^R^ cells (**Figures 1D,E**).

### Furanodiene Depolarized the Mitochondrial Membrane Potential (ΔΨ*m*) in MCF-7/DOX^R^ Cells

**FIGURE 2:**
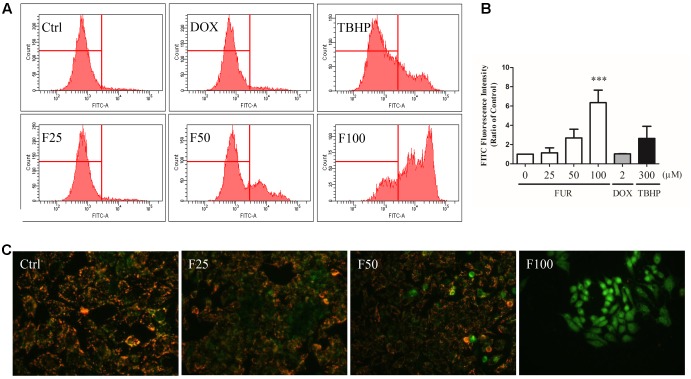
Effect of furanodiene (FUR or F) on ΔΨ*m* in doxorubicin (DOX)-resistant MCF-7 breast cancer cells. **(A)** Cells were treated with furanodiene (0–100 μM), doxorubicin (2 μM), and *tert*-Butyl hydroperoxide (TBHP) (300 μM) for 4 h, after which, the green fluorescence (FITC) from the JC-1 monomers was monitored with JC-1 using flow cytometry. **(B)** Statistical result of **(A)**. **(C)** Cells were exposed to furanodiene (0–100 μM) for 24 h followed by a 30-min incubation with JC-1 monomers. The fluorescence was visualized at 400× magnification using high content imaging. The green fluorescence from the JC-1 monomers was used to represent the cell that lost ΔΨ*m*. TBHP treatment was used as a positive control. Ctrl stands for control. Data are expressed as mean ± SEM. ^∗∗∗^*P* < 0.001 vs. control.

After 4 h of furanodiene treatment in MCF-7/DOX^R^ cells, the JC-1 monomer fluorescence (green) intensity increased dose-dependently, compared with the vehicle control. However, doxorubicin (2 μM) failed to decrease ΔΨ*m* loss in MCF-7/DOX^R^ cells as shown in the flow cytometry results (**Figure 2A**). For example, furanodiene (100 μM) treatment increased JC-1 monomer fluorescence (green) intensity with sixfold, compared with the vehicle control. *tert*-Butyl hydroperoxide (TBHP) (300 μM), as a positive control, also significantly depolarized the mitochondrial membrane potential (**Figure 2B**). Similarly, fluorescent images also showed that furanodiene could decrease the ΔΨ*m* of MCF-7/DOX^R^ cells after a 24-h treatment with furanodiene. The red fluorescence (J-aggregates) intensity is significantly diminished whereas the green fluorescence (monomer) intensity is enhanced after 50 μM treatment with furanodiene. Furthermore, there was only intensive green fluorescence observed after 100 μM treatment with furanodiene, indicating the significant decrease or loss of ΔΨ*m* (**Figure 2C**).

### Furanodiene Regulated the Mitochondrial Pathway of Cell Death in MCF-7/DOX^R^ Cells

**FIGURE 3:**
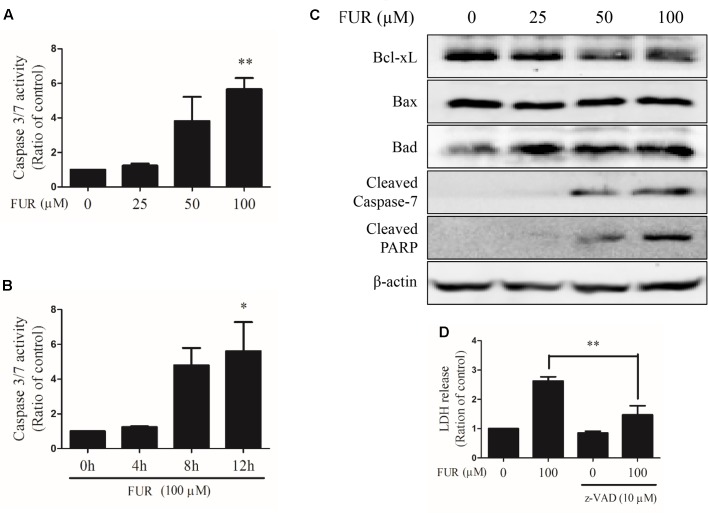
Effect of furanodiene (FUR) on the mitochondrial pathway of apoptosis in doxorubicin-resistant MCF-7 breast cancer cells. **(A)** Cells were treated with different concentrations of furanodiene (0–100 μM) for 24 h. **(B)** Cells were treated with furanodiene (100 μM) for 0–12 h. Caspase activities were determined with Caspase-Glo assay kit. **(C)** Cells were treated with different concentrations of furanodiene (0–100 μM) for 24 h. Protein expression was determined by western blotting and β-actin was used as a loading control. **(D)** Cells were pretreated with z-VAD-fmk (z-VAD) for 1 h before treatment with furanodiene for 24 h. Cytotoxicity was detected by LDH assay. Data are expressed as mean ± SEM. ^∗^*P* < 0.05 and ^∗∗^*P* < 0.01 vs. control.

Compared to vehicle control, furanodiene activated Caspase-3/7 in dose- and time-dependent manners (**Figures 3A,B**). Briefly, 100 μM of furanodiene treatment (24 h) increased the Caspase-3/7 activities with 5.65 folds, compared with the vehicle control. Western blotting results showed that furanodiene treatment decreased Bcl-xL expression, and increased Bad expression, without affecting Bax expression. Meanwhile, furanodiene dose-dependently activated Caspase-7 and PARP, as indicated by the increasing expression of cleaved protein (**Figure 3C**). Moreover, pre-treatment with Caspase inhibitor (z-VAD-fmk) could reverse the cytotoxic effect of furanodiene with a sharp reduction (from 2.62 to 1.47 folds) in MCF-7/DOX^R^ cells (**Figure 3D**). These results indicated that furanodiene might induce cell death through the mitochondrial pathway.

### Furanodiene Induced Apoptotic Cell Death in MCF-7/DOX^R^ Cells

**FIGURE 4:**
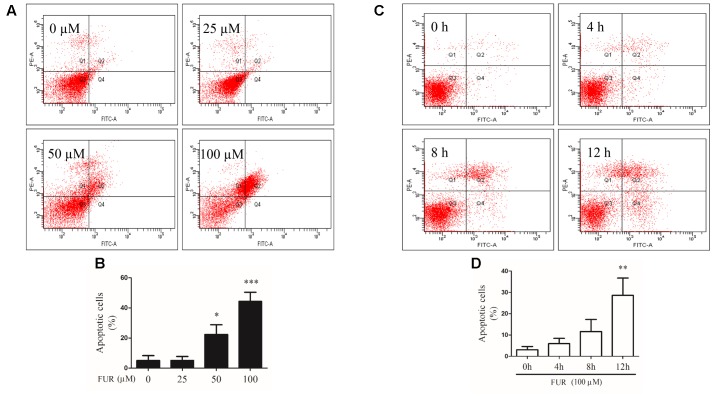
Effect of furanodiene (FUR) on cell apoptosis in doxorubicin-resistant MCF-7 breast cancer cells. **(A)** Cells were treated with different concentrations of furanodiene (0–100 μM) for 24 h. Apoptotic cells were detected by Annexin V/PI staining assay. **(B)** Statistical result of **(A)**. **(C)** Cells were treated with furanodiene (100 μM) for 0–12 h. Apoptotic cells were detected by Annexin V/PI staining assay. **(D)** Statistical result of **(C)**. Data are expressed as mean ± SEM. ^∗^*P* < 0.05, ^∗∗^*P* < 0.01, and ^∗∗∗^*P* < 0.001 vs. control.

To confirm that MCF-7/DOX^R^ cells induced by furanodiene undergo programmed cell death, Annexin V/PI staining was performed using flow cytometry analyses. As shown in **Figures 4A,C**, representative scatter plots of cells showed the differences post treatment with different concentrations of furanodiene used at for different times. The percentage of early/late apoptotic cells was summarized in **Figures 4B,D**. In vehicle control, the proportion of early and late apoptotic cells was 5.07% and 24-h treatments with furanodiene (25–100 μM) dose-dependently increased the ratio of early and late apoptotic cells from 5.10 to 44.27% (**Figure 4B**). Moreover, time-dependent treatments (0–12 h) with furanodiene (100 μM) increased the percentage of early and late apoptotic cells up to 28.57% (**Figure 4D**). The dose- and time-dependent promotion of cell apoptosis induced by furanodiene was also consistent with the outcomes of Caspase-3/7 activation.

### Furanodiene Induced Reactive Oxygen Species (ROS) and Calcium Production in MCF-7/DOX^R^ Cells

**FIGURE 5:**
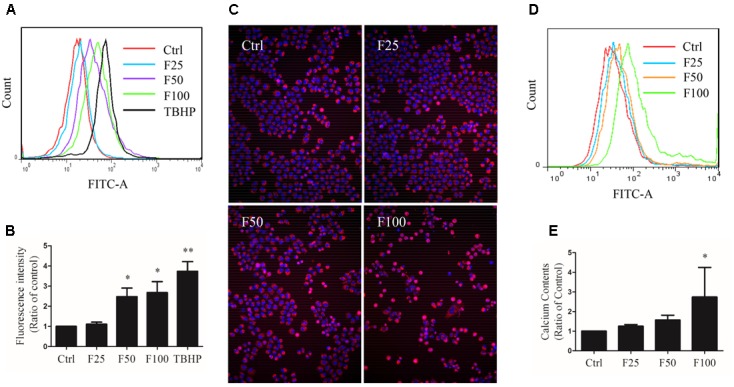
Effect of furanodiene (F) on intracellular reactive oxygen species (ROS) and calcium levels in doxorubicin-resistant MCF-7 breast cancer cells. **(A)** Cells were exposed to furanodiene (0–100 μM) for 2 h after a 30-min pre-incubation with H2DCF-DA. The fluorescence was detected using flow cytometry. tert-Butyl hydroperoxide (TBHP) treatment (300 μM) was used as a positive control. **(B)** Statistical result of **(A)**. **(C)** Cells were exposed to furanodiene (0–100 μM) for 24 h followed by a 30-min incubation with CellROX Deep Red reagent and Hoechst 33342. The fluorescence was visualized at 100× magnification using high content imaging. **(D)** Cells were exposed to furanodiene (0–100 μM) for 2 h after a 30-min pre-incubation with Fluo-4 AM. The fluorescence was detected using flow cytometry. **(E)** Statistical result of **(D)**. Ctrl stands for control. Data are expressed as mean ± SEM. ^∗^*P* < 0.05 and ^∗∗^*P* < 0.01 vs. control.

Cells were exposed to TBHP and furanodiene for 2 h, and ROS production was detected with H2DCF-DA probe using flow cytometry. As shown in **Figures 5A,B**, TBHP (300 μM) treatment induced ROS generation in MCF-7/DOX^R^ cells, and furanodiene treatment dose-dependently induced the ROS generation. Long-term effects of furanodiene on ROS levels was investigated with the CellROX^®^ Deep Red probe. Similarly, fluorescence microscopy results confirmed that furanodiene could dose-dependently increase the ROS generation of MCF-7/DOX^R^ cells after a 24-h treatment with furanodiene (**Figure 5C**). However, addition of NAC (5 mM) or GSH (5 mM) did not attenuate cell viability induced by furanodiene (data not shown). These results indicated that furanodiene inducing cell death might be involved more than ROS-mitochondrial dependent pathway. Moreover, calcium production was also induced by furanodiene (**Figures 5D,E**).

### Furanodiene Regulated the Extrinsic Apoptosis Pathway in MCF-7/DOX^R^ Cells

**FIGURE 6:**
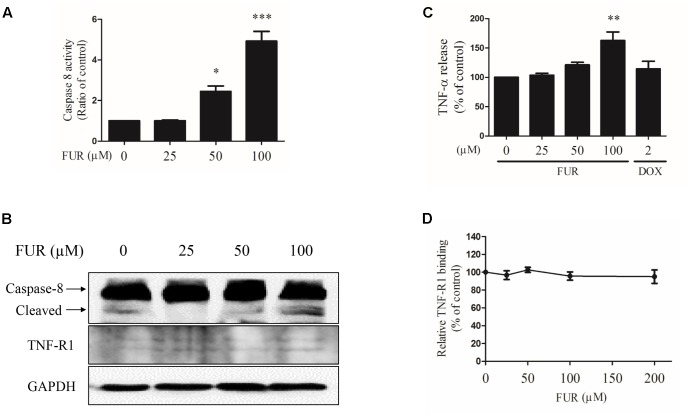
Effect of furanodiene (FUR) on the extrinsic pathway to trigger apoptosis in doxorubicin (DOX)-resistant MCF-7 breast cancer cells. Cells were treated with different concentrations of furanodiene (0–100 μM) or doxorubicin (2 μM) for 24 h. **(A)** Caspase activities were determined with Caspase-Glo assay kit. **(B)** Protein expression was determined by western blotting and GAPDH was used as a loading control. **(C)** Cells were treated with different concentrations of furanodiene (0–100 μM) or doxorubicin (2 μM) for 4 h. TNF-α release was determined with ELISA kit. **(D)** Microtiter plates coated with TNF-α were incubated with TNFR-1 together with furanodiene at the indicated concentrations (0–200 μM). TNF-R1 binding was detected using anti-TNF-R1 antibody and horseradish peroxidase-conjugated secondary antibody. Data are expressed as mean ± SEM. ^∗^*P* < 0.05, ^∗∗^*P* < 0.01, and ^∗∗∗^*P* < 0.001 vs. control.

**FIGURE 7:**
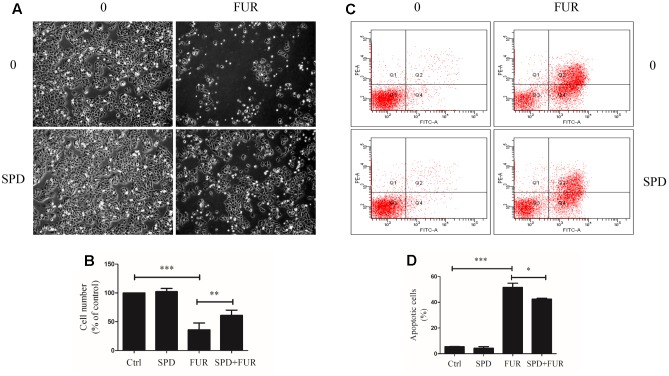
Effect of furanodiene (FUR) on TNF-α pathway trigger to cell death in doxorubicin-resistant MCF-7 breast cancer cells. Cells were pretreated with SPD304 (SPD, 5 μM) for 1 h before treatment with 100 μM of furanodiene for 24 h. **(A)** The morphology alteration and cell number were visualized using microscope at 100× magnifications. **(B)** Statistical result of **(A)**. **(C)** Apoptotic cells were detected by Annexin V/PI assay. **(D)** Statistical result of **(C)**. Ctrl stands for control. Data are expressed as mean ± SEM. ^∗^*P* < 0.05, ^∗∗^*P* < 0.01, and ^∗∗∗^*P* < 0.001 vs. control.

Compared to vehicle control, furanodiene treatment promoted the Caspase-8 activities in a dose-dependent manner (**Figure 6A**). Additionally, western blotting results also confirmed that Caspase-8 cleavage was induced by furanodiene (**Figure 6B**). Furthermore, furanodiene treatment induced a substantial TNF-α release from MCF-7/DOX^R^ cells up to 1.5 folds compared with unstimulated cells. Meanwhile, no significant activation of TNF-α release was observed after doxorubicin treatment alone (**Figure 6C**). As shown in **Figure 6B**, furanodiene did not affect the protein expression of TNF-R1. A binding assay was performed to show that furanodiene was not a specific inhibitor of TNF-α-TNF-R1 binding (**Figure 6D**). Then, a TNF-α antagonist SPD304 was used to examine furanodiene-induced apoptotic cell death. Results showed that addition of SPD304 significantly rescued the survivability (**Figures 7A,B**) and attenuated the cell apoptosis (**Figures 7C,D**) of MCF-7/DOX^R^ cells induced by furanodiene (100 μM). These findings suggest that furanodiene induces cell apoptosis through activating the extrinsic apoptosis pathway via inducing TNF-α release and activating Caspase-8.

### Furanodiene Regulated the NF-κB Pathway in MCF-7/DOX^R^ Cells

**FIGURE 8:**
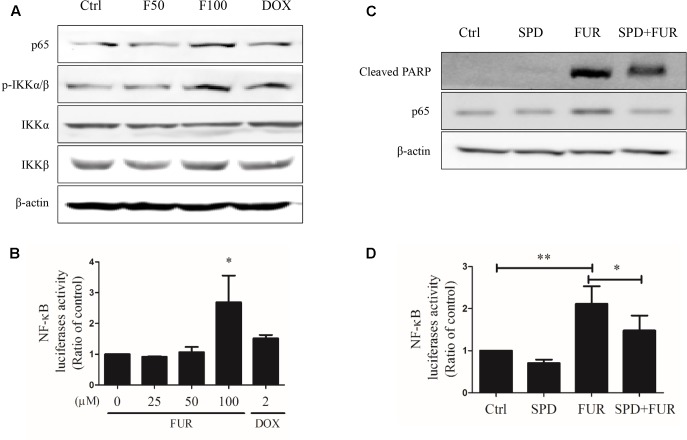
Effect of furanodiene (FUR or F) on NF-κB pathway in doxorubicin (DOX)-resistant MCF-7 breast cancer cells. **(A)** Cells were treated with different concentrations of furanodiene (0–100 μM) or doxorubicin (2 μM) for 24 h. Protein expression was determined by western blotting and β-actin was used as a loading control. **(B)** Cells transfected with NF-κB luciferase reporter gene were exposed to different concentrations of furanodiene (0–100 μM) or doxorubicin (2 μM) for 24 h. NF-κB transcription activity was detected with the dual-luciferase reporter system. **(C)** Cells were pretreated with SPD304 (SPD, 5 μM) for 1 h before treatment with furanodiene (100 μM) for 24 h. Protein expression was determined by western blotting and β-actin was used as a loading control. **(D)** Cells transfected with NF-κB luciferase reporter gene were pretreated with SPD304 (5 μM) for 1 h before treatment with furanodiene (100 μM) for 24 h. NF-κB transcription activity was detected with the dual-luciferase reporter system. Ctrl stands for control. Data are expressed as mean ± SEM. ^∗^*P* < 0.05 and ^∗∗^*P* < 0.01 vs. control.

Furanodiene induced a substantial TNF-α release from MCF-7/DOX^R^ cells, which could likely elicit TNF-α activation of downstream in cell apoptosis. Western blotting results showed that furanodiene increased p65 expression and induced IKKα/β phosphorylation, without changing the protein expression levels of IKKα or IKKβ with significance in MCF-7/DOX^R^ cells (**Figure 8A**). MCF-7/DOX^R^ cells were transiently co-transfected with the NF-κB-responsive luciferase reporter gene and the *Renilla* luciferase control reporter gene. Results showed that furanodiene (100 μM) treatment increased NF-κB transcriptional activity of the transfected cells up to 2.70 folds compared with vehicle control (**Figure 8B**). However, doxorubicin (2 μM) treatment just slightly induced NF-κB-driven luciferase reporter activity and IKKα/β phosphorylation without significantly affecting the protein expression levels of p65, IKKα, and IKKβ. Addition of SPD304 significantly attenuated PARP activation, p65 protein expression, and NF-κB transcriptional activity of transfected MCF-7/DOX^R^ cells induced by furanodiene (100 μM) (**Figures 8C,D**).

### Knockdown of NF-κB/p65 Attenuated the Pro-apoptotic Effects of Furanodiene

**FIGURE 9:**
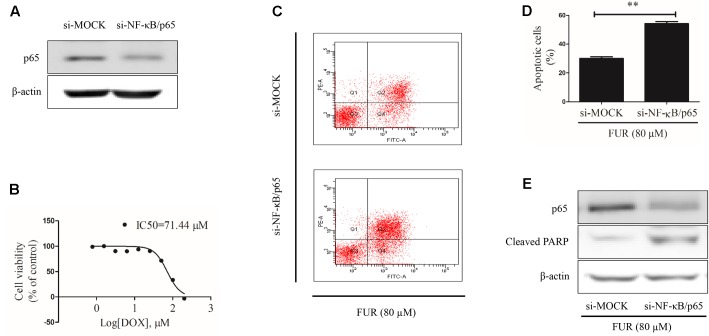
Effect of furanodiene (FUR) on cell apoptosis in NF-κB/p65 knockdown doxorubicin (DOX)-resistant MCF-7 breast cancer cells. **(A)** Cells were transfected with siRNAs using Lipofectamine 2000. The transfection efficacy of siRNAs was confirmed by western blotting. **(B)** NF-κB knockdown cells were exposed to different concentrations of doxorubicin for 48 h. Cell viability was detected by MTT assay. **(C)** Transfected cells were exposed to furanodiene (80 μM) for 24 h. Apoptotic cells were detected by Annexin V/PI assay. **(D)** Statistical result of **(C)**. **(E)** Cells were treated with furanodiene (80 μM) for 24 h after transfection with siRNAs. Protein expression was measured by western blotting and β-actin was used as a loading control. Data are expressed as mean ± SEM. ^∗∗^*P* < 0.01 vs. control.

At the start of this experiment, western blotting results showed that p65 expression was knocked down in MCF-7/DOX^R^ cells transfected with si-NF-κB/p65 compared with si-MOCK. Notably, knockdown of p65 expression could not affect β-actin expression (**Figure 9A**) and p65-knockdown cells were still resistant to doxorubicin, with an IC50 value of 71.44 μM (**Figure 9B**). Annexin V/PI staining results confirmed that knockdown of p65 expression significantly enhanced the pro-apoptotic effect of furanodiene (**Figures 9C,D**). Furanodiene could not induce p65 expression up-regulation, but enhanced PARP activation in MCF-7/DOX^R^ cells transfected with si-NF-KB/p65, compared with the cells transfected with si-MOCK (**Figure 9E**).

## Discussion And Conclusion

**FIGURE 10:**
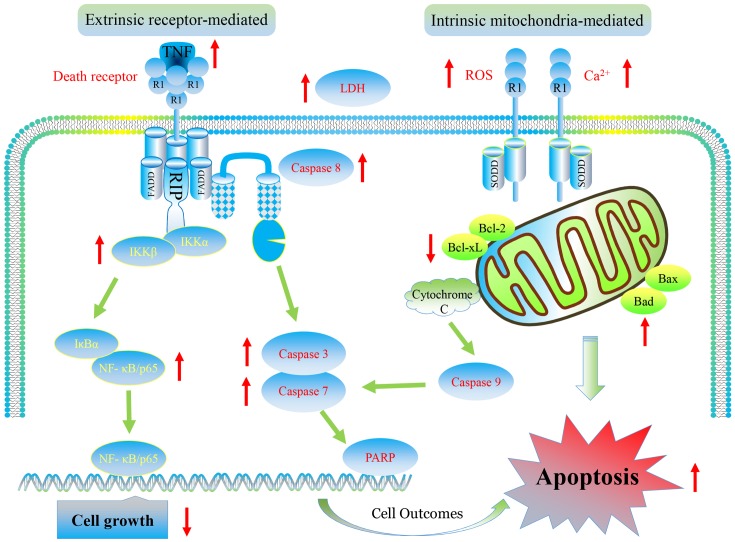
Graphic representation of pathways for furanodiene-induced cell apoptosis and for doxorubicin-resistant MCF-7 cells outcomes. Furanodiene preferentially causes cancer cell death through inducing cell apoptosis via extrinsic- and intrinsic-dependent pathways and NF-κB-independent pathway in doxorubicin-resistant MCF-7 breast cancer cells.

Our results show that furanodiene preferentially causes cancer cell death through inducing cell apoptosis via extrinsic- and intrinsic-dependent pathways and NF-κB-independent pathway in doxorubicin-resistant breast cancer cells (**Figure 10**). The intrinsic apoptosis pathway is triggered by furanodiene, as demonstrated by alteration of the mitochondrial membrane potential, decrease of anti-apoptotic regulators such as Bcl-xL, increase of pro-apoptotic regulators such as Bad, activation of Caspase-7 and PARP, and promotion of ROS and calcium generation. Inhibition of caspase activations by inactivating caspase components significantly abolish furanodiene-induced cell death. Unexpectedly, addition of anti-oxidants cannot attenuate cell viability induced by furanodiene. We speculate that furandiene also induces cell apoptosis through activation of other pathways, so we focused on the extrinsic apoptosis pathway induced by furanodiene. Our results show that furanodiene-induced cell apoptosis is correlated with activation of Caspase-8 and accumulation of TNF-α release, and is not directly associated with TNF-R1 expression and TNF-α/TNF-R1 binding. Furthermore, addition of TNF-α antagonist significantly rescued cell survival and attenuated cell apoptosis induced by furanodiene. These observations suggest that furanodiene functions on cell death, at least in part, by interfering with the intrinsic and extrinsic apoptosis pathways.

The extrinsic apoptosis pathway is triggered by a death ligand binding to a death receptor, such as TNF-α to TNFR. Previous research identified that the binding of TNFR and TNF-α activates NF-κB pathway, which favored both cell survival and apoptosis, depending on the cell type and biological context (Hehlgans and Pfeffer, 2005). In our study, furanodiene treatment increases NF-κB transcriptional activity in MCF-7/DOX^R^ cells, and addition of TNF-α antagonist significantly attenuates NF-κB transcriptional activity induced by furanodiene. We can conclude that NF-κB activation is potentially attributed to furanodiene-induced TNF-α increase in MCF-7/DOX^R^ cells. Many studies have explored the relationship between NF-κB expression and prognosis in solid tumors, but the conclusion remains elusive. Wu’s group performed a meta-analysis to demonstrate that NF-κB expression has strong connections with poor prognosis in most solid tumors irrespective of NF-κB localization (Wu et al., 2015). Interestingly, furanodiene treatment also significantly increases the NF-κB/p65 expression and IKKα/β phosphorylation. Knockdown of NF-κB/p65 significantly sensitizes MCF-7/DOX^R^ cells to furanodiene on cell apoptosis, indicating that furanodiene-induced cell apoptosis is dependent on TNF-α pathway and independent on NF-κB pathway. It also suggests that NF-κB inhibitors can be used to enhance furanodiene-induced cancer cell death. Previous studies indicated that doxorubicin induced drug resistance via up-regulating NF-κB expression in MCF-7 human breast cancer cells (Fang et al., 2014), and many agents targeting NF-κB exerted anti-tumorigenic activity in doxorubicin-resistant MCF-7 breast cancer cells (Tran et al., 2013; Meiyanto et al., 2014). For instance, curcumin and mollugin acquire reversal of chemo-resistance by inhibiting the NF-κB signaling pathway. In contrast, furanodiene failings to inhibit the NF-κB pathway may be attributed to TNF-α activation in the doxorubicin-resistant MCF-7 cells. Therefore, furanodiene, could be combined with NF-κB inhibitors (from natural products or chemical synthesis) to overcome chemo-drug resistance.

Our findings prompt that furanodiene displays anti-cancer effects through inducing cell apoptosis via targeting multiple signaling pathways and may be developed as a promising natural product for multidrug-resistant cancer therapy in the future.

## Author Contributions

Z-FZ designed and conducted the experiments, and wrote the manuscript. H-BY and D-QL designed the experiments and revised the manuscript. C-MW, W-AQ, S-PW, J-MZ, HY, LC, and TW gave some data interpretations and provided valuable feedback to this conception. Y-TW organized, conceived, and supervised the study. All authors read and approved the manuscript.

## Conflict of Interest Statement

The authors declare that the research was conducted in the absence of any commercial or financial relationships that could be construed as a potential conflict of interest.
