# Corrigendum: Furanodiene Induces Extrinsic and Intrinsic Apoptosis in Doxorubicin-Resistant MCF-7 Breast Cancer Cells via NF-κB-Independent Mechanism

**DOI:** 10.3389/fphar.2017.00934

**Published:** 2017-12-18

**Authors:** Zhang-Feng Zhong, Hai-Bing Yu, Chun-Ming Wang, Wen-An Qiang, Sheng-Peng Wang, Jin-Ming Zhang, Hua Yu, Liao Cui, Tie Wu, De-Qiang Li, Yi-Tao Wang

**Affiliations:** ^1^Guangdong Key Laboratory for Research and Development of Natural Drugs, School of Public Health, Guangdong Medical University, Zhanjiang, China; ^2^State Key Laboratory of Quality Research in Chinese Medicine, Institute of Chinese Medical Sciences, University of Macau, Macao, China; ^3^Division of Reproductive Science in Medicine, Department of Obstetrics and Gynecology, Feinberg School of Medicine, Northwestern University, Chicago, IL, United States; ^4^Center for Developmental Therapeutics, Chemistry of Life Processes Institute, Northwestern University, Evanston, IL, United States; ^5^Department of Pharmacy, The Second Hospital of Hebei Medical University, Shijiazhuang, China

**Keywords:** furanodiene, multidrug resistance, breast cancer, cell apoptosis, intrinsic/extrinsic apoptosis, NF-κB

In the published article, there was an error in affiliation 1. Instead of “^1^Guangdong Key Laboratory for Research and Development of Natural Drugs, Guangdong Medical University, Zhanjiang, China”, it should be “^1^Guangdong Key Laboratory for Research and Development of Natural Drugs, School of Public Health, Guangdong Medical University, Zhanjiang, China.” The authors apologize for this error and state that this does not change the scientific conclusions of the article in any way.

The original article has been updated.

## Conflict of interest statement

The authors declare that the research was conducted in the absence of any commercial or financial relationships that could be construed as a potential conflict of interest.

